# Sucralose: From Sweet Success to Metabolic Controversies—Unraveling the Global Health Implications of a Pervasive Non-Caloric Artificial Sweetener

**DOI:** 10.3390/life14030323

**Published:** 2024-02-29

**Authors:** José Alfredo Aguayo-Guerrero, Lucía Angélica Méndez-García, Helena Solleiro-Villavicencio, Rebeca Viurcos-Sanabria, Galileo Escobedo

**Affiliations:** 1 Laboratory of Immunometabolism, Research Division, General Hospital of Mexico “Dr. Eduardo Liceaga”, Mexico City 06720, Mexico; jose.aguayo01@iest.edu.mx (J.A.A.-G.);; 2 Posgrado en Ciencias Genómicas, Autonomous University of Mexico City, Mexico City 06720, Mexico; 3 Department of Plastic and Hand Surgery, Medical Center-University of Freiburg, 79106 Freiburg, Germany

**Keywords:** sucralose, non-caloric artificial sweeteners, T1R3, microbiota, liver damage, insulin resistance, FAS, proinflammatory cytokines

## Abstract

Sucralose is a food additive initially used to mitigate glycemic peaks and calorie intake in patients with diabetes and obesity. Although sucralose has been considered safe for human consumption, the World Health Organization (WHO) issued a global alert in 2023 concerning the potential health implications of this artificial sweetener. This review aims to comprehensively explore the effects of sucralose intake on human health by understanding sucralose absorption, metabolism, and excretion. We also outline the role of the sweet taste 1 receptor 3 (T1R3) in mediating sucralose-dependent signaling pathways that regulate satiety, incretin release, and insulin response. Finally, we discuss the impact of sucralose on microbiome dysbiosis, inflammatory response origin, liver damage, and toxicity. Gaining a deeper understanding of the manifold effects of sucralose on human physiology will help promote further studies to ensure its consumption is deemed safe for a broader population, including children, adolescents, and pregnant women.

## 1. Introduction

Sucralose, a prevalent food additive, is utilized to impart sweetness to foods without contributing a substantial caloric load [1]. It dominates the global sweetener market, comprising 30% of the United States sweetener market [2]. Present in over 4500 food and beverage items, sucralose plays a pivotal role in the food industry and is projected to strengthen its market presence [2,3].

Discovered in 1976 by Shashikant Phadnis, sucralose, chemically identified as 1,6-dichloro-1,6-dideoxy-β-D-fructofuranosyl-4-chloro-4-deoxy-α-D-galactopyranoside, is an artificial sweetener derived from sucrose [4,5]. Produced through selective halogenation, involving the addition of three chlorine atoms to the sucrose molecule, this process diminishes intestinal absorption [5].

Originally intended for individuals with obesity and diabetes due to its ability to avert glycemic spikes and reduce caloric intake while maintaining sweetness [6], sucralose has become a ubiquitous sweetener across diverse demographics. Boasting unique characteristics such as prolonged solid-state stability and resilience in solutions with varying pH levels [7], it remains 600 times sweeter than regular sugar, with a bacteriostatic effect that prevents dental caries [8,9]. For years, sucralose has been regarded as a harmless substance. In 1998, the Joint Committee of Experts on Food Additives of the WHO/FAO within the United Nations classified this sweetener as a safe product [10]. The FDA has also stated that sucralose is safe for consumption by children and individuals with diabetes [11]. The WHO has reported an acceptable daily intake for this sweetener at 15 mg/kg of body weight [12]. Sucralose initially appeared to offer a fresh solution to the rising rates of obesity and metabolic diseases [13]. However, despite the apparent harmlessness of sucralose, the World Health Organization (WHO) has recently issued an alert indicating that sucralose consumption may be linked to systemic inflammation and metabolic diseases [14].

## 2. Sucralose Metabolism

Sucralose constitutes a modified sucrose molecule with three chlorine atoms replacing three hydroxyl groups [13]. This minor structural alteration induces substantial functional changes (Table 1). Notably, sucralose exhibits poor absorption (only 16%) in the small intestine compared to glucose and fructose [13].

The journey of dietary sugar from the intestinal lumen to enterocytes involves the Na+/glucose cotransporter, specifically known as sodium–glucose-linked transporter 1 (SGLT1) [15]. Sweet taste receptor type 1 member 3 (T1R3) and the taste G protein gustducin, present in enteroendocrine cells, detect sweet molecules like sucrose or sucralose in the intestines [15]. This detection triggers an upsurge in the expression of SGLT1, facilitating glucose absorption.

Sucralose undergoes elimination from the human body primarily through feces, with the remaining portion excreted in the urine. As per previous studies, only 16% of it is absorbed in the upper gastrointestinal tract [7,13]. While a considerable amount of sucralose is eliminated unchanged, recent research has unveiled the presence of conjugated glucuronide metabolites of sucralose in waste material [7]. Additionally, reports indicate acetylated sucralose metabolites in rat waste [16]. These findings underscore the need to investigate the impact of sucralose metabolites.

It is noteworthy that sucralose can persist in the bloodstream for over 18 h post-ingestion [2]. Furthermore, observations indicate that this sweetener can traverse the placenta, reaching the fetus in pregnant women, and is also present in breast milk [2,17]. Concerns regarding the duration of sucralose in the body have been raised by researchers. A study by Bornemann et al., involving rats consuming sucralose for 40 days, reported the detection of acetylated metabolites in urine up to 11 days after the last dose [18]. The study also revealed sucralose presence in rat adipose tissue up to two weeks post the final dose [18], presenting results contradictory to prior data. Hence, further research is imperative to comprehend the persistence of sucralose and the effects of its metabolites in the body.

Contrary to expectations, the bioavailability of sucralose in the human body varies with age. Sylvetsky et al. determined that children consuming a 300 mL diet drink exhibit plasma concentrations of sucralose twice as high as adults [19]. Sylvetsky suggests that this may be attributed to children’s lower glomerular filtration rate.

## 3. T1R3: The Sucralose Binding Receptor

The perception of sweet taste is mediated by the G protein-coupled receptor located in the taste buds of the oral cavity [20]. This receptor constitutes a heterodimer formed by T1R3 and the taste receptor type 1 member 2 (T1R2) [20]. Notably, the sweet taste receptor is expressed not only in taste buds, but also in various tissues including the brain, pancreas, small intestine, and colon [16,20]. Generally, non-caloric artificial sweeteners (NCAS) interact with the T1R family of sweet taste receptors via the G protein associated with α-gustducin, leading to elevated intracellular cAMP levels and increased neurotransmitter release [21].

**Figure 1:**
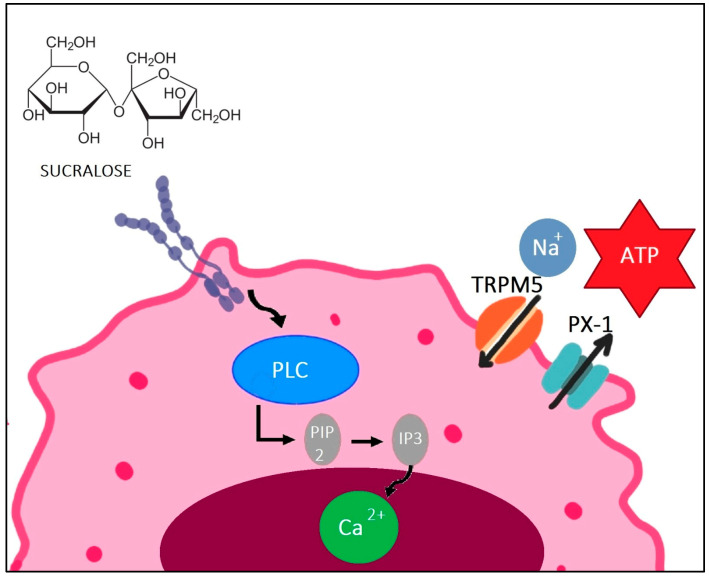
Molecular mechanism of sucralose’s interaction with T1R3 Receptor. The figure illustrates the intricate process by which sweet-tasting molecules such as sucralose bind to the T1R3 sweet taste receptor. This receptor is not only present in taste buds, but is also distributed in various tissues, including the brain, intestine, pancreas, and liver. Upon binding to the receptor, phospholipase C is activated, leading to the conversion of phosphatidylinositol bisphosphate inositol trisphosphate. This activation triggers the release of calcium, opening TRPM5 channels, allowing sodium influx. Finally, the opening of pannexin 1 channels leads to the release of ATP into the synaptic cleft, stimulating efferent nerve fibers. This intricate cascade of events highlights the diverse roles of the T1R3 receptor beyond its classical involvement in taste perception. Abbreviations: PLC, phospholipase C; PIP2, phosphatidylinositol bisphosphate; IP3, inositol trisphosphate; Ca^2^+, calcium; TRPM5, transient receptor potential cation channel subfamily M member 5; Na+, sodium; PX1, pannexin 1 channel; ATP, adenosine triphosphate.

Specifically, sucralose binds to its receptor on taste bud cells, inducing the dissociation of the G protein, α-gustducin. This event initiates the activation of phospholipase C, which cleaves phosphatidylinositol 4,5-bisphosphate (PIP2) into inositol 1,4,5-triphosphate (IP3). IP3, in turn, stimulates calcium release, activating the transient receptor potential cation channel subfamily M member 5 (TRPM5) channel, facilitating Na+ entry. Consequently, the pannexin 1 channel opens, releasing ATP into the synaptic cleft. This intricate process stimulates nerve-efferent fibers, transmitting the sensation of sweetness [22] (Figure 1). Sugars and sweeteners like sucralose, acesulfame K, and sucrose bind to the T1R2 and T1R3 sites of sweet taste receptors on the tongue. Conversely, other sweeteners like aspartame exclusively bind to T1R2 [23].

The T1R2 + T1R3 sweet taste receptor combination detects luminal glucose concentration within enteroendocrine cells. When the threshold is exceeded, the taste signaling pathway (comprising T1R2 + T1R3 and α-gustducin) activates. Consequently, there is an upsurge in the production of incretins such as glucose-dependent insulinotropic polypeptide (GIP) and glucagon-like peptide-1 (GLP-1) [15]. These hormones regulate appetite, intestinal motility, and insulin secretion [24], with GLP-1 directly influencing the hypothalamus, promoting the sensation of satiety [25] and reducing gastric motility, delaying gastric emptying and thereby prolonging the feeling of fullness [25].

Within the gastrointestinal system, a pathway like the one in the mouth is activated, resulting in the positive regulation of SGLT1 (which transports glucose from the intestine to enterocytes) [16]. GLUT2 is subsequently activated, facilitating the mobilization of glucose into the bloodstream [16].

As mentioned earlier, both sucralose and sucrose bind to the T1R2/T1R3 receptor; however, the effects produced by these molecules differ significantly. Sugar, being a hydrophilic molecule, is poorly absorbed by taste buds [10]. In contrast, sucralose, a hydrophobic molecule with a robust sweet taste, is more effectively absorbed by taste buds. Moreover, in T1R3-knockout mice, the preference for sucralose is eliminated, whereas the preference for sugar only decreases [26]. Functional brain imaging in humans further reveals distinctions between these two molecules, with sucrose eliciting a stronger brain response in the anterior insula, frontal operculum, striatum, and anterior cingulate compared to sucralose, activating the dopaminergic areas of the mesencephalon [27].

There is also information indicating that the consumption of sucralose by women and individuals with obesity generates a stronger response in the neuronal reward system [28]. Pertinent to this, the role of a recently discovered cell type, neuropod cells, comes to light. These intestinal sensory epithelial cells allow the transmission of signals from the intestinal environment to the brain [29].

In both humans and animals, the preference for conventional sugar over NCAS is precisely linked to neuropods [30]. In the gut, neuropods synapse with vagal sugar neurons to transmit stimuli to the brain. Cholecystokinin (CCK)-labeled duodenal neuropod cells transduce stimuli from sweeteners and sugars to the vagus nerve using sweet taste receptors and sodium–glucose transporters [30].

The two types of sugar stimulation elicit different neuronal pathways, with sweeteners stimulating purinergic neurotransmission, and conventional sugar stimulating glutamatergic neurotransmission [30]. In summary, the preference for sugar over sweeteners in mice depends on the glutamatergic signaling of neuropod cells [30].

## 4. Effect of Sucralose Consumption on Satiety

The literature provides insights into the impact of sucralose consumption on satiety. Firstly, Wang et al. conducted a study on Drosophila melanogaster flies, investigating the long-term effects of sucralose on energy homeostasis [31]. Flies fed with sucralose exhibited chronic sweet imbalance, leading to hyperactivity, insomnia, glucose intolerance, and hyperphagia. These effects were reversible upon sucralose removal, and the response was mediated by neuropeptide F (NPF), the fly equivalent of mammalian neuropeptide Y (NPY), known to stimulate appetite. The study also revealed that sucralose consumption triggered a hypothalamic response to fasting through AMPK activation, indicating that chronic sweet consumption activates a conserved neuronal starvation response, increasing the motivation to eat [31].

A question arises about whether sucralose alone induces metabolic changes or if it requires a combination with other foods. In a study by Sánchez-Tapia et al. on rats, a diet containing sucralose increased the secretion of incretins such as GIP and GLP-1 [32]. When combined with a high-fat diet, the effect was amplified, resulting in higher levels of GIP and GLP-1 and a decrease in GLUT-4 levels in white adipose tissue, leading to hyperglycemia. Sucralose also increased the expression of taste receptors T1R2 and T1R3 in the tongue and epithelium of the small intestine, resulting in hyperinsulinemia [32].

Geraedts et al. studied intestinal secretin tumor cells (STC-1) in the presence of sucralose and found higher levels of CCK and GLP-1. Combining pea protein with sucralose further increased CCK and GLP-1 levels, suggesting a synergistic effect when combining dietary elements [33]. Human duodenal tissue stimulated with sucralose plus pea protein also exhibited elevated CCK and GLP-1 levels [33].

CCK and GLP-1 exert multiple effects on appetite, including enhancing the sensation of satiety and delaying gastric emptying [34]. In contrast, GIP’s impact on satiety is not as pronounced as that of GLP-1; some studies suggest that it may indirectly influence appetite through its effects on insulin secretion and glucose metabolism [34].

In addition to incretins, leptin, secreted by adipocytes, plays a significant role in the appetite–satiety axis [35]. Prolonged high levels of leptin can lead to leptin resistance, increasing the threshold for feeling full [35]. Kohno et al. discovered that neurons activated by leptin are also stimulated by sucralose, suggesting that sucralose consumption could potentially disrupt the appetite–satiety axis and raise the threshold for feeling full [36]. Velázquez et al. reported that sucralose consumption in rats increased the expression of ΔFosB in dopaminergic brain nuclei, promoting food intake and suggesting a potential link between sucralose consumption and dysregulation of neural mechanisms controlling food intake [37].

## 5. Metabolic Effects of Sucralose Consumption

Sucralose has been identified as an activator of sweet taste receptors in pancreatic β-cells and enteroendocrine cells, leading to the increased secretion of crucial hormones, including incretins and insulin [38]. Recent studies highlight the necessity of direct carbohydrate exposure to the small intestine mucosa for the secretion of GLP-1 and GIP [39].

The sweet taste receptors of mouse enteroendocrine cell lines (GLUTag and STC-1) are colocalized with GLP-1 and GIP [26], which could explain the increase in these incretins when stimulated with sucralose. Human, rodent, and in vitro studies show that sucralose can alter glucose, insulin, and GLP-1 levels [40,41]. In this sense, Hamano et al. corroborated these results in in vitro studies with pancreatic MIN6 cells. This group reported that sucralose caused an increase in insulin levels [42], and when using lactisole to block the T1R3 receptor, the secretion of insulin produced by the sweetener was inhibited [42].

In a study by Kundu et al., human adipose tissue-derived mesenchymal stromal cells (MSCs) were investigated in vitro to assess the impact of sucralose [43]. The study demonstrated an elevation in reactive oxygen species (ROS) after three days of sucralose stimulation, suggesting a dose-dependent increase in adipogenesis in these cells [43]. These findings align with those observed in mice by Qing Shi et al., where sucralose exposure led to elevated sweet taste receptor expression and lower glucose tolerance, possibly due to increased glucose transporters [20].

In a study by Ragi et al., rats fed with sucralose for seven weeks showed an increase in weight and body fat until the third week of treatment, indicating that changes induced by sucralose are not immediate but occur with constant exposure [6]. Qian et al. revealed that lower sucralose doses improved glucose intolerance, but higher doses over time increased GLP-1 and serum glucose levels in both rats and humans [44]. Similar observations in animal and in vitro models have been documented in healthy humans [41].

The impact of sucralose consumption is influenced by the health condition of individuals. Sylvetsky et al. experimented on overweight women, revealing changes in NF-κB inflammatory pathways in subcutaneous adipose tissue biopsies after eight weeks of consuming light beverages [45]. Notably, these changes were also observed in young and healthy individuals by Dalenberg et al., where decreased insulin sensitivity occurred with sucralose-containing diet drinks paired with a carbohydrate-rich diet [46]. Brown et al. studied individuals with type 1 diabetes (DMT1), finding increased GLP-1 secretion after glucose load followed by sucralose–acesulfame diet beverage consumption [47]. Gómez-Arauz et al. reported an increase in cellular markers of systemic inflammation, blood glucose, and insulin levels in healthy young adults consuming a daily amount of sucralose equivalent to that found in a 500 mL “light” drink [48]. These findings were consistent with Pepino et al.’s study on obese subjects [49]. Lertrit et al. observed lower insulin sensitivity and acute insulin response in healthy subjects given a 200 mg sucralose pill for four weeks, although there was an increase in GLP-1 release [50]. Similarly, Romo-Romo et al. found insulin resistance during glucose tolerance tests in healthy subjects receiving 15% of the allowed sucralose intake for 14 days [41]. Despite existing evidence on the metabolic effects of sucralose, some results remain controversial. For example, Temizkan et al. reported contradictory effects in healthy subjects and patients with newly diagnosed T2DM, suggesting that measuring the acute effect of sucralose may not reflect its long-term outcomes [51].

The mode of sucralose administration influences its impact on energy metabolism. In a study by Jin Ma et al., intragastric sucralose administration (80 and 800 mg in 500 mL saline solution) showed no release of insulin, GLP-1, or GIP, indicating limited benefits for dietary control in T2DM patients [26]. Early-age sucralose consumption may increase the preference for sweetened beverages later in life, leading to weight gain and reduced secretion of GIP associated with insulin secretion, as well as a decrease in the HOMA index [52]. Reports suggest that sucralose can reduce humoral immunity in Peyer’s patches and a percentage of IgA plasma cells, but it increases the humoral response in the lamina propria by elevating the number of B cells, IgA, and IL-4 [52].

## 6. Sucralose Consumption and Changes in Microbiota

The interaction between NACS and the gut microbiome can result in notable metabolic effects on the host, even in the absence of eukaryotic recognition and metabolism [53]. Recent studies suggest that sucralose consumption may lead to dysbiosis, adversely affecting the composition of intestinal microbiota. Short-chain fatty acids (SCFAs), derived from our diet, bind with G protein-coupled receptors (GPCRs) in the gastrointestinal tract [21]. A reduction in bifidobacteria, coupled with an increase in enterobacteria, results in chronic low-grade inflammation associated with conditions such as insulin resistance and heightened intestinal permeability [21]. Dai et al. observed signs of intestinal inflammation and reduced butyrate-producing bacteria in the offspring of female mice fed sucralose during pregnancy and lactation [54]. Additionally, sucralose has been linked to an increased mutation rate of *E. coli*, promoting resistance to potent antibiotics like rifampicin [55]. Changes in the microbiota are known contributors to inflammation development. In a study by Bian et al., rats exposed to sucralose for six months exhibited altered intestinal microbiota, increased proinflammatory gene activity in the liver, and elevated levels of metalloproteinase 2 (MMP-2), TNF-alpha, IL-6, and iNOS [56]. The heightened MMP-2 and iNOS levels are associated with increased activity of TNF-α and IL-1β, which are classical proinflammatory cytokines [56]. The study also identified higher levels of the *Ruminococcaceae Ruminococcus* bacterium in the sucralose group, which is commonly found in individuals with Crohn’s disease (CD) [56]. Furthermore, the sucralose group displayed increased multidrug resistance genes, reduced secondary bile acids with antimicrobial functions, and alterations in tryptophan metabolism leading to an increase in pro-inflammatory quinolinic acid and a decrease in anti-inflammatory and neuroprotective kynurenic acid [56]. In mouse models of CD, sucralose was found to promote the development of intestinal proteobacteria [57].

In a study by Zheng et al., mice exposed to different doses of sucralose (0.0003, 0.003, 0.03, and 0.3 mg/mL) for 16 weeks showed an increase in Tenacibaculum, Ruegeria, Staphylococcus, and Allobaculum in the jejunum, ileum, and colon compared to the control group [58]. Additionally, sucralose consumption in humans for ten weeks resulted in an increase in the relative abundance of Blautia coccoides and a decrease in Lactobacillus acidophilus compared to the control group [59]. Recent research suggests that changes in the microbiota may impact the offspring. During the final trimester of pregnancy, there is a transfer of bacteria from the mother’s intestinal tract to breast milk, facilitated by various immune system cells [60]. Sucralose has the potential to alter the composition of the maternal intestinal microbiota, and consequently, this could affect breast milk during the bacterial transfer process. Indeed, according to a report by Tapia-González and their team, women with high consumption of NCAS, including sucralose, exhibit an increased presence of the unclassified archaea Methanobrevibacter spp. in colostrum [61]. A previously established link connects the heightened presence of this archaeon with obesity in Mexican children [61].

## 7. Sucralose Consumption and Inflammation

Wang et al. assert that sucralose increases the risk of colitis in rats [62]. In their study, the consumption of the sweetener was associated with higher body weight, increased disease activity, elevated serum D-lactic acid levels, and higher levels of TNF-α and IL-1β in intestinal tissue compared to control rats. The same research group reported that sucralose heightened the number and size of colorectal tumors induced by azoxymethane and sodium dextran sulfate in mice [63]. In the sucralose group, inflammatory pathways (TNFα, IL-1β, IL-6, and TLR4/Myd88/NF-κB signaling) and the STAT3/VEGF tumor-associated signaling pathways were increased [63]. Additionally, both Bian et al. and Rodriguez-Palacios et al. reported that sucralose could exacerbate intestinal inflammatory activity in mice at risk of Crohn’s disease [56,57]. Due to potential intestinal dysbiosis, it is believed that sucralose could be a primary contributor to inflammatory bowel disease (IBD) [64].

The intestine possesses a protective layer composed of mucin (85% carbohydrate side chain) [62]. Intestinal bacteria play a significant role in degrading this mucus layer by breaking down the carbohydrate side chains of mucin. Therefore, altering the proportions of the microbiota can be harmful and trigger inflammatory bowel disease [65].

Dai et al. conducted a study in mice that were administered sucralose only during pregnancy and lactation [58]. At 12 weeks of age, the mice were sacrificed, and a higher hepatic expression of IL-6 and TNF-alpha was reported compared to the control group.

The inflammatory consequences of sucralose consumption can persist across generations, as shown in the study by Aguayo-Guerrero et al. [66]. This group fed human mothers either with a high sucralose intake (HSI) of more than 36 mg sucralose/day during pregnancy, or a low sucralose intake (LSI) of less than 60 mg sucralose/week. They demonstrated that newborns from the HSI mothers showed a substantial increase in their percentage of inflammatory nonclassical monocytes compared to neonates from the LSI mothers. Additionally, the umbilical cord tissue of infants from HSI mothers exhibited higher IL-1 beta and TNF-alpha levels along with lower IL-10 expression than that found in newborns from LSI mothers. This evidence shows that heavy sucralose ingestion during pregnancy affects the metabolic and inflammatory features of neonates [66].

## 8. Liver Damage and Sucralose Consumption

Studies of rats provide evidence that sucralose can deactivate hepatic ribosomes, leading to cytokine-mediated inflammation in the liver [67]. Furthermore, Dhurandhar et al. examined the effects of sucralose consumption in rats over 30 days [68]. These rats developed hepatic fibrosis, Kupffer cell hyperplasia, and lymphocyte infiltration. Moreover, Farid et al. investigated the impact of sucralose on mice after 8 and 16 weeks of treatment [69]. Sucralose increased Hb1Ac levels, reduced red and white blood cells, and decreased hematocrit and hemoglobin levels [69]. By 18 weeks, markers of liver and kidney function had increased. Subsequent histopathological studies revealed severe liver and kidney damage [69]. Bian et al. conducted a study on rats exposed to sucralose for six months, contributing to the development of liver inflammation [56]. Higher levels of metalloproteinase 2 (MMP-2), TNF-alpha, IL-6, and iNOS were detected in the liver. The elevation of MMP-2 and iNOS is associated with increased activity of TNF-α and IL-1β, which are classically proinflammatory cytokines [56]. Azad et al. investigated the effects of sucralose consumption during pregnancy in female mice and reported that the experimental group exhibited greater weight, increased fat mass, and higher insulin resistance at 12 weeks of age compared to the control group [70].

Dai et al. administered sucralose to mice during pregnancy and lactation [54]. Subsequently, these mice received a high-fat diet from weaning to 12 weeks of age. Mice in the sucralose group developed a higher degree of steatosis compared to the control group. Additionally, these mice displayed higher levels of the FAS protein in the liver.

These data are intriguing because in vitro cultures of HepG2 cells show a decrease in the levels of the hypogenic transcription factor when the T1R3 receptor is blocked, compared to cells cultured in the presence of sucralose and the T1R3 receptor [71].

This indicates that the binding of sucralose to the T1R3 receptor can enhance lipogenic activity in the liver. Furthermore, sucralose has been demonstrated to induce ROS production in the liver through T1R3, thereby facilitating hepatic lipogenesis and contributing to HFD-induced fatty liver [71]. Alyaa F. et al. validated these findings in mice fed with sucralose [69]. Histopathological examination of the livers of mice in sucralose- and stevia-administered groups revealed severe damage in this organ, characterized by the loss of hepatic architecture, with dilated centrals, apoptotic hepatocytes, intra-lobular inflammatory infiltrates, and an area of hemorrhage [69].

Similarly, Meng and colleagues analyzed the liver tissue of five mice exposed to a sucralose diet. They observed that mice fed with sucralose exhibited decreased insulin signal transduction in both HepG2 cells and liver tissue compared to mice fed with a standard chow diet [72]. This diminished insulin signaling subsequently compromised glucose utilization by liver cells, potentially leading to impaired hepatic homeostasis, and triggering anaerobic metabolism with the accumulation of ROS products.

## 9. Sucralose Toxicity

Rahn et al. argue that using sucralose for baking can pose health risks since, when employed at 250 °C in the presence of glycerol or lipids, the metabolites of this sweetener may contribute to the formation of toxic chloropropanols [73]. Furthermore, this sweetener releases HCl when heated to 119 °C [74]. Dong et al. report that sucralose promotes the formation of dioxin-like polychlorinated biphenyls (dl-PCBs) during cooking at 160 °C [75].

According to Oliveira et al., working with sucralose at high temperatures is hazardous. At 125 °C, the crystal structure of sucralose changes, giving rise to harmful polychlorinated aromatic hydrocarbons (PCAH) [76]. Similarly, Dong et al. found that heating sucralose in stainless steel at high temperatures induces a chlorination reaction, resulting in the production of toxic compounds such as polychlorinated dibenzo-p-dioxins and dibenzofurans (PCDD/Fs) [77]. These compounds were detected in the smoke produced during the cooking process and in the residues.

It is important to note that sucralose is not only present in food and drinks, but also in other products such as electronic cigarettes. These devices contain sweetening liquids (e-liquid) that contain sucralose. Even small amounts of sucralose (0.05% mole) in e-liquids can increase the production of aldehydes (carbonyls) and hemiacetals, which could impact long-term health [78]. Pasqualli et al. state that sucralose is cytotoxic, genotoxic, and immunotoxic [79]. This working group cultured lymphocytes and demonstrated that sucralose reduces these cells. They reported an increase in the damage to the genetic material and structural changes in the chromosomes of the lymphocytes. They propose that sucralose could modulate gene expression, especially by interfering with the MAPK8 (cell proliferation), APTX (DNA repair), and EID1 (histone regulation) genes [79].

Several years ago, concerns were raised about the potential carcinogenic effects of consuming sucralose. However, subsequent studies have produced mixed results. In a study conducted by Soffritti et al., mice fed with sucralose from birth to natural death exhibited an increase in hematopoietic neoplasms [80]. Nevertheless, the data obtained by Soffritti et al. was later rejected by the Panel on Food Additives and Nutrient Sources Added to Food (ANS) [81].

More recently, Debras et al. conducted a study on a cohort of 102,865 French adults, revealing that the consumption of aspartame and acesulfame-K is associated with an overall increased risk of cancer [82]. However, no association between sucralose and cancer was found. The authors noted that this could be due to the lower exposure to sucralose as compared to aspartame and acesulfame-K [82].

Studies on mice have shown that high sucralose intake adversely affects the immune system by interfering with T-cell activity [83]. Sucralose alters the structure of T-cell membranes, leading to reduced effectiveness in T-cell receptor signaling and intracellular calcium release [84]. Furthermore, a sucralose metabolite known as sucralose-6-acetate has been found in rodent feces and identified as genotoxic [84]. This compound increases the expression of genes associated with inflammation, oxidative stress, and cancer, especially the MT1G gene. It also impacts the integrity of the intestinal barrier and the permeability of the human colon [84]. Additionally, it inhibits two enzymes from the cytochrome P450 family, CYP1A2 and CYP2C19 [84].

All this evidence presented together highlights the toxic role of sucralose. This involves detrimental consequences of sweetener intake across different domains of health and emphasizes the importance of avoiding consumption to prevent harmful effects. Moreover, the World Health Organization (WHO) recently recommended against the use of non-sugar sweeteners and also warned that long-term use of sweeteners might have potentially undesirable effects [14].

## 10. Conclusions

**Figure 2:**
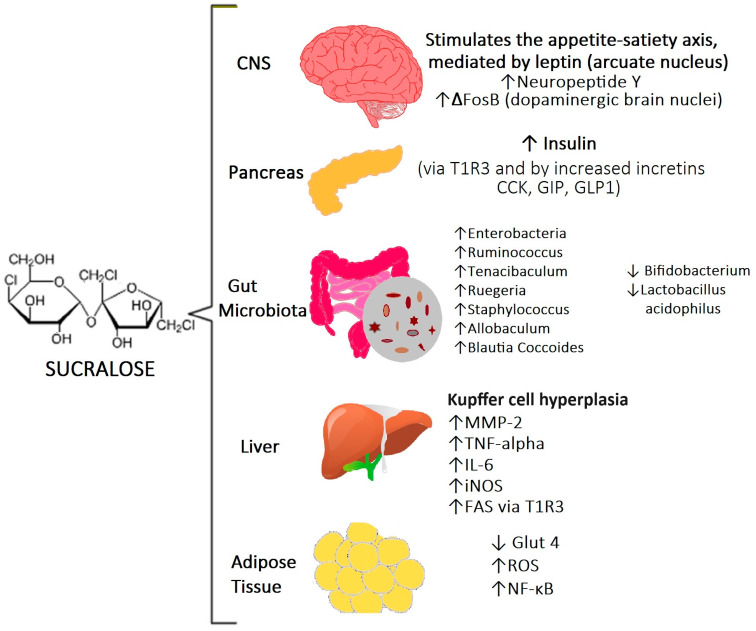
In the CNS, sucralose is reported to potentially interfere with the appetite–satiety axis, while also increasing the expression of neuropeptide Y and ΔFosB. Within the pancreas, sucralose is implicated in elevating insulin levels—mediated through the T1R3 receptor—and the augmentation of incretin secretion. Studies involving both animal models and humans suggest that sucralose consumption disrupts the intestinal microbiota. Furthermore, in the liver, sucralose is associated with an upregulation of the lipogenic pathway via FAS and the T1R3 receptor. In adipose tissue, it downregulates Glut 4 levels, increases ROS, and activates the NF-κB inflammatory pathway. Abbreviations: T1R3, taste receptor type 1 member 3; CCK, cholecystokinin; GIP, gastric inhibitory polypeptide; GLP1, glucagon-like peptide 1; MMP-2, matrix metalloproteinase-2; iNOS, nitric oxide synthases; FAS, fatty acid synthase; ROS, reactive oxygen species; NF-κB, nuclear factor kappa-light-chain-enhancer of activated B cells; CNS, central nervous system.

The consumption of sucralose, a commonly used artificial sweetener, is associated with various adverse health effects. Despite being considered safe following previous studies, recent research suggests possible links to systemic inflammation, metabolic diseases, disruptions in gut microbiota, liver damage, and toxic effects at the cellular level (Table 2, Figure 2).

It is crucial to highlight the persistence of sucralose in the body, its ability to cross the placenta, and its presence in breast milk, raising concerns about prenatal and neonatal exposure. Additionally, the variability of sucralose absorption and metabolism in different population groups, including children and adults, is emphasized. The present review provides evidence of the metabolic and microbiome effects associated with the consumption of sucralose. These findings should be considered when formulating public policies regarding the compound. However, the data presented have a few limitations that need to be taken into account. Firstly, most of the studies have been conducted in animal models. Secondly, the clinical studies have heterogeneous designs, sample sizes, and populations, which precludes the possibility of drawing general conclusions. Therefore, more studies are needed to establish the safety and efficacy of sucralose.

The interaction of sucralose with sweet taste receptors (T1R2/T1R3) and its effects on cell signaling, as well as its impact on satiety and metabolic regulation, are key aspects that require a deeper understanding. This article consolidates evidence that sucralose consumption can affect gut microbiota, trigger inflammatory responses, and potentially contribute to the development of metabolic diseases such as insulin resistance.

This review also highlights the potential toxicity of sucralose, especially when exposed to high temperatures during cooking processes, and its presence in products beyond the food realm, such as e-cigarette liquids.

The review underscores the need to reconsider the safety and long-term impact of sucralose consumption, challenging previous claims about its safety. It offers a comprehensive view of the potential risks associated with this artificial sweetener, which could have significant implications for public health and food policy formulation.

## Figures and Tables

**Table 1:** Differences between sucralose and sugar.

Name	Sucralose	Sucrose
Formula	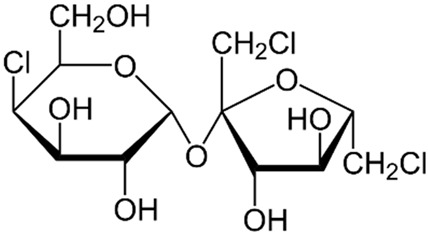	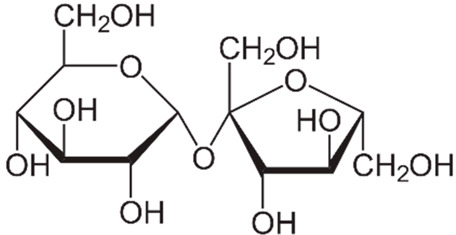
Origin	Artificial	Natural
Molecule	Hydrophobic	Hydrophilic
Binding to the T1R3 receptor in taste buds	Absorbed more intensively by the taste buds.	Poorly absorbed by the taste buds.
Caloric intake	Low	High
Effect on glycemia	It is considered to have a minimal impact on blood glucose levels, but recent evidence has prompted us to reevaluate this aspect.	Increases blood glucose levels.
Metabolism	84% is not absorbed. However, recent evidence suggests the elimination of conjugated glucuronide metabolites of sucralose and the presence of acetylated sucralose metabolites in rat waste.	It metabolizes into glucose and fructose.
Intestinal absorption	16% is absorbed at the intestinal level.	Completely absorbed.
Sweetness	600 times sweeter than sucrose.	Sweetness = 1.
Neuronal reward system	Higher response	Lower response
Vagal sugar neurons in the gut	Purinergic neurotransmission	Glutamatergic neurotransmission

**Table 2:** Sucralose experimental models.

Model	Effect	Ref.
STC-1 cells	↑ CCK, ↑ GLP-1	Geraedts (2012) [33]
MIN6 cells	↑ Insulin	Hamano (2015) [42]
Enteroendocrine L cells of mice	↑ GLP1, ↑ GLP2	Romo-Romo (2018) [41]
Mesenchymal stromal cells (human)	↑ ROS↑ Adipogenesis	Kundu (2020) [43]
Lymphocytes	Interferes MAPK8 (cell proliferation), APTX (DNA repair) and EID1 (histone regulation), ↓ lymphocytes	Pasqualli (2020) [79]
D. melanogaster	Hyperactivity, insomnia, glucose intolerance, hyperphagia.	Wang (2016) [31]
Rats	↑ GIP, ↑ GLP1, ↑ T1R2 and ↑ T1R3 in tongue and epithelium of the small intestine.↓ GLUT 4 in white adipose tissue, hyperinsulinemia	Sánchez-Tapia (2019) [32]
Rats	↑ ΔFosB in dopaminergic brain nuclei.↑ Food intake	Velazquez (2020) [37]
Rats	↑ Weight, ↑ body fat	Ragi (2021) [6]
Rats and humans	↑ GLP-1, ↑ glucose	Qian (2021) [44]
Rats	↑ Ruminococcaceae Ruminococcus bacterium, ↑ multidrug resistance genes, ↓ secondary biliary acids with antimicrobial functions. ↑ quinolinic acid (proinflammatory), ↓ kynurenic acid (anti-inflammatory and neuroprotective)	Bian (2017) [56]
Rats	↑ Risk of colitis, ↑ disease activity, ↑ body weight, ↑ serum D lactic acid, ↑ TNF-a and IL-1b in intestinal tissue.	Wang (2019) [62]
Rats	↑ Number and size of colorectal tumors, splenomegaly, ↑ inflammatory pathways (TNF-a, IL-1B, IL-6, TLR4, MYd88, NFkB), ↑ tumor-associated signaling pathways (STAT3/VEGF)	Li (2020) [63]
Rats	Hepatic fibrosis, Kupffer cell hyperplasia, lymphocyte infiltration.	Dhurandhar (2018) [68]
Mice models of CD	↑Intestinal proteobacteria, ↑ MPO	Rodriguez-Palacios (2018) [57]
Mice (sucralose during gestation and lactation)	↓ butyrate producing bacteria. Hepatic steatosis, ↑ FAS	Dai (2020) [54]
Mice (sucralose during gestation)	↑ Weight, ↑ fat mass, ↑ insulin resistance	Azad (2020) [70]
Mice	↑ Tenacibaculum, Ruegeria, Staphylococcus, and Allobaculum in the jejunum, ileum, and colon	Wu (2022) [71]
Mice	↑ Hb1Ac, ↓ red and white blood cells, ↓ hematocrit, ↓ hemoglobin.	Farid (2020) [69]
Overweight women with no history of light beverages	↑ NF-kB	Sylvetsky (2020) [45]
Young healthy subjects	↓ Insulin sensitivity ↓	Dalenberg (2020) [46]
Humans with DM1	↑ GLP-1	Brown(2012) [47]
Healthy young adults	↑ Glucose, ↑ insulin, ↑ cellular markers of inflammation.	Gómez-Arauz (2019) [48]
obese subjects	↑ Glucose, ↑ insulin, ↑ cellular markers of inflammation.	Pepino(2013) [49]
Healthy subjects	↓ Insulin sensitivity, ↓ AIR, ↑ GLP-1	Lertrit (2018) [50]
Healthy subjects	Insulin resistance	Romo-Romo (2018) [41]
